# A 44-kb deleted-type copy number variation is associated with decreasing complement component activity and calf mortality in Japanese Black cattle

**DOI:** 10.1186/s12864-021-07415-6

**Published:** 2021-02-06

**Authors:** Shinji Sasaki, Youko Miki, Takayuki Ibi, Hiroyuki Wakaguri, Yuichi Yoshida, Yoshikazu Sugimoto, Yutaka Suzuki

**Affiliations:** 1 University of the Ryukyus, Faculty of Agriculture, 1 Senbaru, Nishihara, Nakagami-gun, Okinawa, 903-0213 Japan; 2 United Graduate School of Agricultural Sciences, Kagoshima University, 1-21-24 Korimoto, Kagoshima, Kagoshima 890-0065 Japan; 3 Hokubu Agricultural Technology Institute, Hyogo Prefectural Technology Center for Agriculture, Forest and Fisher, Asago, Hyogo Japan; 4 Graduate School of Environmental and Life Science, Okayama University, Tsushima-naka, Okayama, 700-8530 Japan; 5 Department of Medical Genome Sciences, and Department of Computational Biology, Graduate School of Frontier Sciences, The University of Tokyo, Chiba, 277-8562 Japan; 6 Shirakawa Institute of Animal Genetics, Japan Livestock Technology Association, Yushima, Bunkyouku, Tokyo, 113-0034 Japan

**Keywords:** Postnatal mortality, Copy number variation, Complement, Beef cattle

## Abstract

**Background:**

Calf mortality generally occurs in calves prior to weaning, which is a serious problem in cattle breeding. Several causative variants of monogenic Mendelian disorders in calf mortality have been identified, whereas genetic factors affecting the susceptibility of calves to death are not well known. To identify variants associated with calf mortality in Japanese Black cattle, we evaluated calf mortality as a categorical trait with a threshold model and performed a genome-wide copy number variation (CNV) association study on calf mortality.

**Results:**

We identified a 44-kb deleted-type CNV ranging from 103,317,687 to 103,361,802 bp on chromosome 5, which was associated with the mortality of 1–180-day-old calves. The CNV harbored *C1RL*, a pseudogene, and an *IncRNA* localized in the *C1R* and *C1S* gene cluster, which is a component of the classical complement activation pathway for immune complexes for infectious pathogens. The average complement activity in CNVR_221 homozygotes at postnatal day 7 was significantly lower than that of wild-type animals and heterozygotes. The frequency of the risk allele in dead calves suffering from diarrhea and pneumonia and in healthy cows was 0.35 and 0.28, respectively (odds ratio = 2.2, *P* = 0.016), suggesting that CNVR_221 was associated with the mortality of Japanese Black calves suffering from an infectious disease.

**Conclusions:**

This study identified a deleted-type CNV associated with the mortality of 1–180-day-old calves. The complement activity in CNVR_221 homozygotes was significantly lower than that in heterozygotes and wild type animals. The frequency of the risk allele was higher in dead calves suffering from an infectious disease than in healthy cows. These results suggest that the existence of CNVR_221 in calves could be attributed to a reduction in complement activity, which in turn leads to susceptibility to infections. Thus, the risk allele could serve as a useful marker to reduce the mortality of infected Japanese Black calves.

**Supplementary Information:**

The online version contains supplementary material available at 10.1186/s12864-021-07415-6.

## Background

Calf health is one of the most important components in cattle production, both in terms of animal welfare and profitability. In both dairy and beef cattle, however, calf mortality, which generally ranges from 2 to 15%, mainly occurs in calves prior to weaning [1–4]. In Japanese Black cattle, the calf mortality rate during the first 180 days, at which point the calves are weaned, is 3.3% (16,292 dead animals/year) [5]; this has mainly been attributed to gastrointestinal and respiratory infectious diseases, such as diarrhea and pneumonia [6]. Recently, several studies discovered a causal variant of single gene disorders (monogenic Mendelian disorders) in calf mortality using single-nucleotide polymorphism arrays and next-generation sequencing technologies [7–12], whereas genetic factors affecting calves’ susceptibility to disease and infections and their association with calf mortality are unknown. Information on the molecular mechanisms underlying the role of infectious diseases in calf mortality provides clues both for the treatment of the disease and breeding, thereby effectively leading to improved calf health.

Copy number variation (CNV) is defined as deletion or duplication regions of the genome that range from 1 kb to several Mb, as reviewed in [13]. CNVs cause diseases through various mechanisms, such as gene dosage alternation and gene structure disturbance, as reviewed in [14]. We previously reported genome-wide CNV maps for Japanese Black cattle [15] and found a CNV related to embryonic mortality in Japanese Black cattle [16]. Thus, the CNV maps also enabled us to search the CNVs associated with the mortality of Japanese Black calves.

In current study, we evaluated calf mortality as a categorical trait with a threshold model [17, 18] and performed a genome-wide CNV association study using these traits. We identified a deleted-type CNV that was associated with decreased complement component activity and calf mortality at the first 180 days (6 months) after birth.

## Results and discussion

### CNVR_221 on chromosome 5 was associated with calf mortality in Japanese Black cattle

The mortality of 1–180-day-old Japanese Black calves was previously estimated from 40,412 calf records in a threshold model using Bayesian analysis with Gibbs sampling [18–20]. The mortality rate of 1–180-day-old calves was 10.33%, and the estimates of direct heritability of calf mortality were 0.165 [18].

**Fig. 1:**
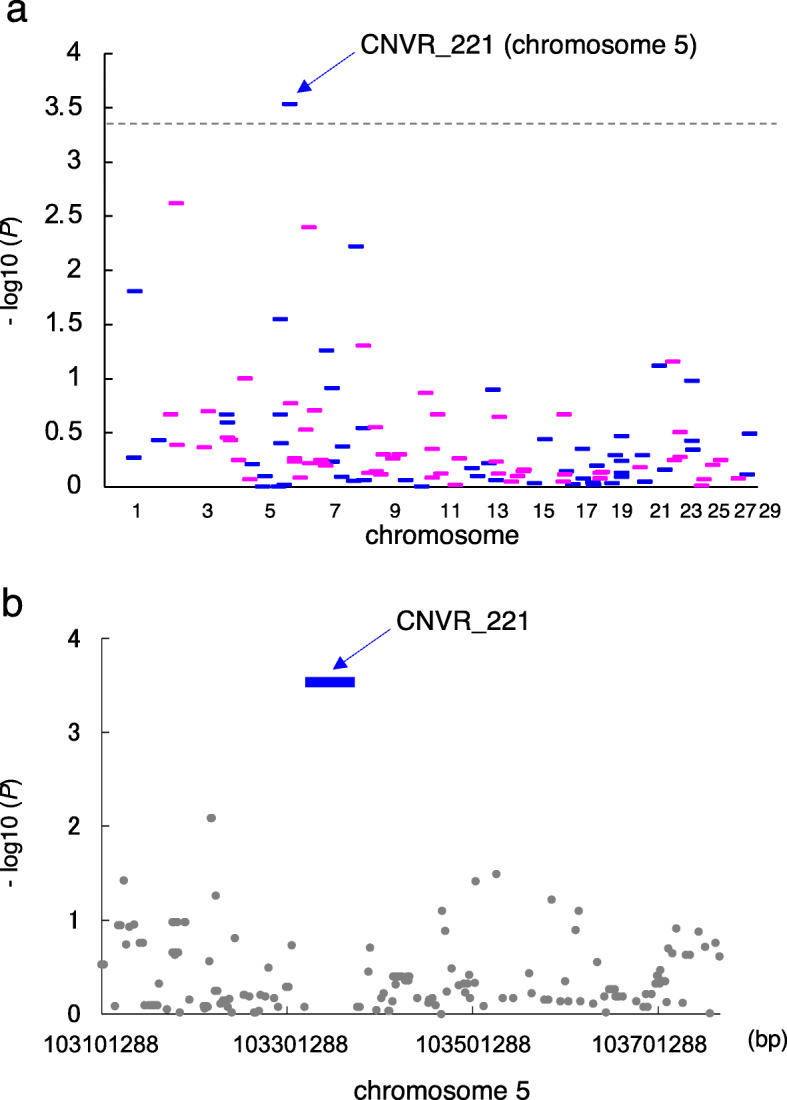
The association of CNVRs with calf mortality in 791 Japanese Black cattle. **a** Manhattan plot of the association of 116 deleted-type CNVRs with the breeding value of calf mortality (calves that died aged 1–180 days) of 791 cows . The blue are odd chromosomes and the red are even chromosomes. The dashed line is the Bonferroni-corrected threshold for genome-wide significance [−log10 (*P*) = 3.365]. **b** Manhattan plot of the association of the surrounding SNPs at an approximate ±200 kb distance from CNVR_221. Positions are based on the ARS-UCD1.2 assembly of the bovine genome

We detected 861 CNV regions (CNVRs), defined as the union area of overlapping CNVs identified in at least two animals [21], in the autosomes of 1481 Japanese Black cattle [15]. Of the 1481 animals, 791 cows were evaluated for the breeding value of calf mortality (calves that died aged 1–180 d) (Additional file 1). Previous reports have indicated that deleted-type CNVs are connected to diseases in cattle [22–26]; thus, in this study, we evaluated the association between deleted-type CNVRs and calf mortality. Of the 861 CNVRs, 116 deleted-type CNVRs (Additional file 2: Table S1), with a minor allele frequency greater than 0.01 in 1481 animals [15] (Additional file 2: Table S1), were used in the association study. To identify CNVR associated with calf mortality, we performed a genome-wide CNV association study with the breeding value of calf mortality of 791 cows, in GEMMA software [27]. We found that CNVR_221 was associated with calf mortality, reaching the Bonferroni-corrected threshold (*P* < 4.31 × 10 ^− 4^, Fig. 1a). CNVR_221 was located within a 44,115 bp window from 103,317,687 to 103,361,802 bp on bovine chromosome 5 (*P* = 2.94 × 10^− 4^) (Table 1). Of note, the surrounding SNPs at a distance of approximately ±200 kb from CNVR_221 were not associated with the traits studied (Fig. 1b) and were not in linkage disequilibrium (LD) with CNVR_221 (Additional file 2: Table S2).

**Table 1:** CNVR_221 with genome-wide significant associations with calf mortality, 0–180 days after birth

CNVR_ID	CNVR_type	Chr	Start (bp)	End (bp)	CNVR_length (bp)	*P*-value
CNVR_221	loss	5	103,317,687	103,361,802	44,115	2.94E-04

SNP positions are based on the ARS-UCD1.2 assembly of the bovine genome.

**Fig. 2:**
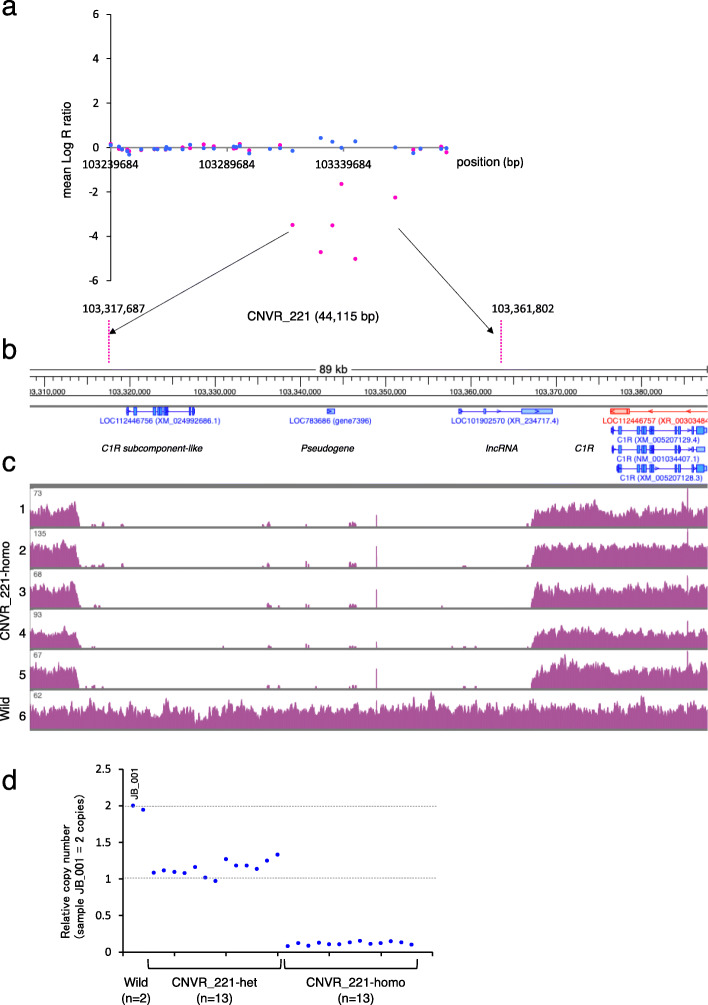
CNVR_221 overlapping with *C1RL*, a *pseudogene* and an *IncRNA* on chromosome 5. **a** Regional SNP plot of CNVR_221. The mean log R ratio is indicated on the *y*-axis. The mean log R ratio of CNVR_221 animals (magenta) and the mean log R ratio of non-CNVR_221 animals (blue) were calculated from 31 animals. SNP positions were based on the ARS-UCD1.2 assembly of the bovine genome. **b** Transcripts in the CNVR_221 region were visualized using Custom Genome Browser. The RefSeq ID and gene symbol were labeled. **c** Alignment of sequence read by WGS derived from 5 CNVR_221 homozygotes and a wild-type animal. **d** qPCR confirmation of CNVR_221. The leftmost bar represents a calibrator animal (JB_001), which is assumed to contain two copies of the DNA. The *x*-axis represents the animals

The mean log R ratio of six SNPs, which were continuously located within a 44,115 bp window between 103,317,687 bp and 103,361,802 bp on chromosome 5, was low (Fig. 2a). Whole genome resequencing and quantitative polymerase chain reaction (PCR) (qPCR) were conducted to verify CNVR calls using the SNP array as two independent experimental validations. Alignment of sequence reads resulted in the disappearance of read counts in the CNVR_221 region from five CNVR_221 homozygotes (Fig. 2c). The copy number calculated by qPCR was approximately one and zero in the CNVR_221 heterozygotes and the homozygotes, respectively (Fig. 2d), which concurred with the expected copy number estimated by the SNP array.

We found that CNVR_221 harbored three transcripts: *C1RL* (complement C1r subcomponent: *LOC112446756*), pseudogene (*LOC783686*), and IncRNA (*LOC101902570*) (Fig. 2b, Additional file 2: Table S3). CNVR was localized on the *C1R* and *C1S* gene cluster (Fig. 2b, Additional file 2: Table S3), which is a component of the classical complement activation pathway for immune complexes for infectious pathogens. C1R and C1S have two CUB, a single EGF, and two CCP modules followed by a serine protease domain [28] (Additional file 2: Table S4). The structure of C1RL (complement C1r subcomponent like: *LOC112446756, XM_024992686.1* was the Refseq in model) was similar to that of C1R, whereas C1RL had no CCP modules and serine protease domain (Additional file 2: Table S4). Although the expression of the *C1RL* transcript was confirmed by RNAseq data (Additional file 3) [29], it is uncertain whether C1RL is a functional protein involved in complement activity.

### CNVR_221 created a *C1RL* (*Complement C1r subcomponent like: LOC112446756*) null allele

**Fig. 3:**
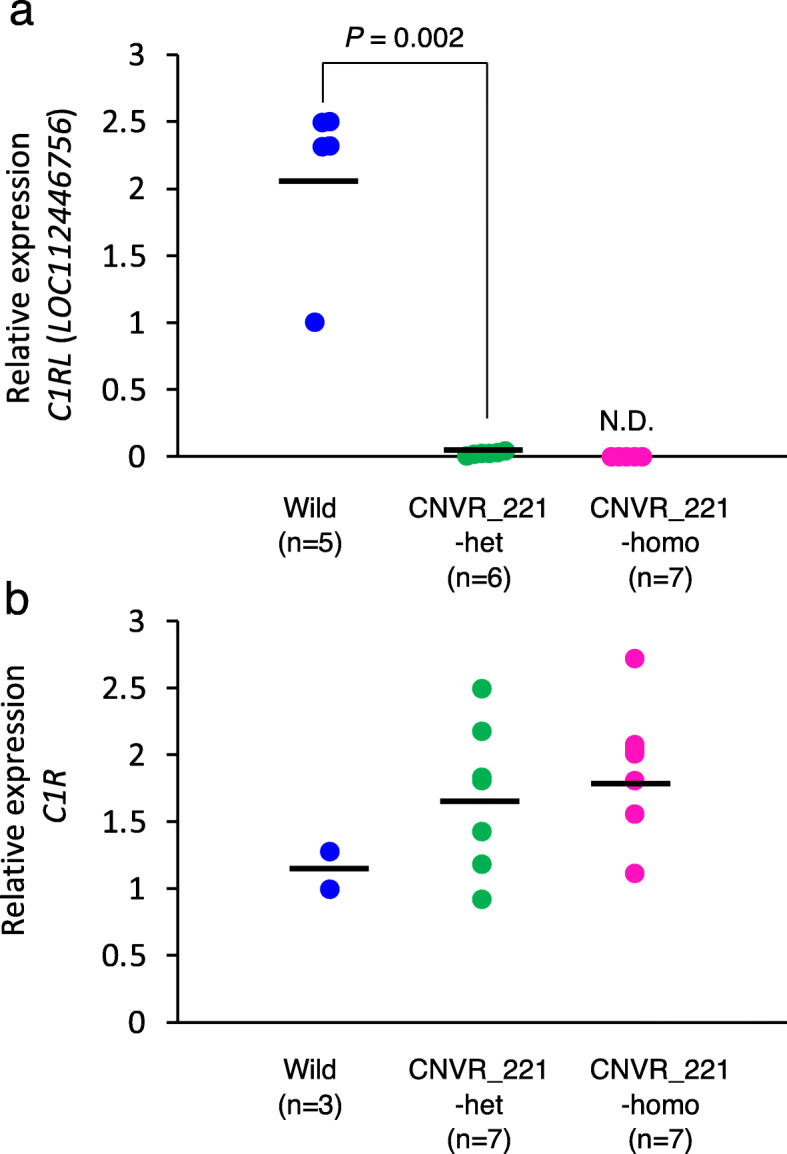
*C1RL* (*LOC112446756*) and *C1R* expression in the liver of wild-type animals, CNVR_221 heterozygotes, and homozygotes. **a** The relative expression of *C1RL* (*LOC112446756*) mRNA in the liver of wild type animals (*n* = 5), CNVR_211 heterozygotes (*n* = 6), and homozygotes (*n* = 7) is plotted on the Y-axis. Bars represent the mean obtained in triplicate from three independent experiments. *P* values determined by *t*-tests are shown. N. D.; not detected. **b** The relative expression of *C1R* mRNA in the liver of wild type animals (*n* = 3), CNVR_211 heterozygotes (*n* = 7), and homozygotes (*n* = 7) is plotted on the Y-axis. Bars represent the mean obtained in triplicate from three independent experiments

CNVR_221, which contained the full length of *C1RL* (*LOC112446756*) (Fig. 2b), resulted in the *C1RL* null allele (Fig. 2c, d). To examine the effect of the 44 kb deletion on the expressions, we conducted RT-qPCR using total RNA from the livers of cattle, as complement genes are mainly expressed in the liver [30–32]. The level of expression in the heterozygous animals was significantly lower than that in the wild-type animals (*P* = 0.002) (Fig. 3a). The expression in the homozygous animals was undetectable (Fig. 3a), suggesting that the *C1RL* (*LOC112446756*) mRNA was expressed in the liver of wild-type animals and *C1RL* (*LOC112446756*) mRNA derived from the CNVR_221 allele could be not expressed; thus, CNVR_221 generated null alleles of *C1RL* (*LOC112446756*).

*C1R* was closely located on the telomeric side at a distance of approximately 15 kbp from CNVR_221 (Fig. 2b), which could include regulatory elements of *C1R* expression. The level of expression in the CNVR_221 homozygotes was not significantly different from that in heterozygotes and wild-type animals (Fig. 3b), indicating that CNVR_221 did not affect *C1R* expression.

### The loss of *C1RL* (*LOC112446756*) in calves decreased the complement activity

C1r and C1s, the serine protease subunit, are components of the classical complement activation pathway, reviewed in [33]. The activation of the classical pathway is primed by antigen-antibody complexes. The complexes trigger the assembly and activation of the C1 complex, C1q–C1r–C1s, causing the activation of the C1r and C1s. Following the activation of the components, later components of the complement cascade from the membrane attack complex (MAC), which creates membrane pores for cell lysis and facilitates phagocytosis through opsonization. Previous studies in humans reported that *C1r* and *C1s* deficient patients had recurrent infections, and result in death at juvenile stages due to severe infection [33–36]. These results suggest that the deficiency of *C1RL* (*LOC112446756*) in CNVR_221 calves could be explained by a reduction in complement activity, which in turn leads to recurrent infections.

**Fig. 4:**
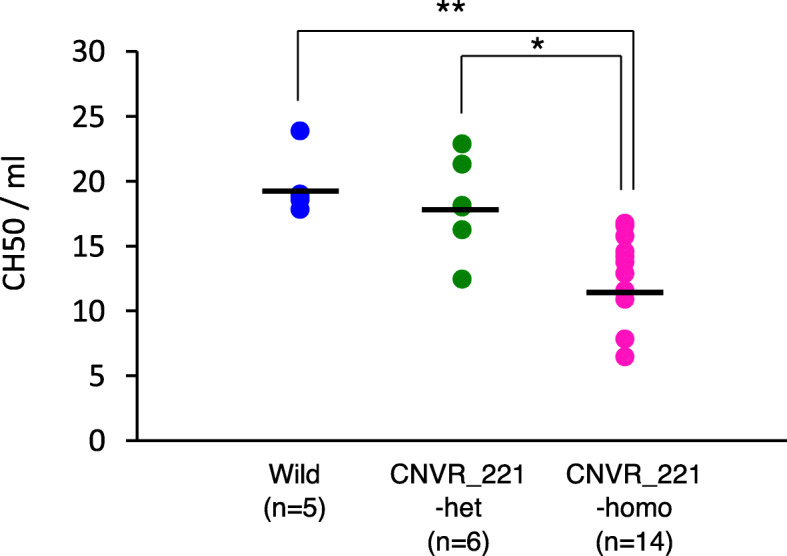
The complement activity in wild type animals, CNVR_221 heterozygotes, and homozygotes. The hemolytic complement activity (CH50 units/mL) in the sera of wild type animals (*n* = 5), CNVR_211 heterozygotes (*n* = 6), and homozygotes (*n* = 14) at 7 days of age are plotted on the Y-axis. The CNVR_211 genotype is indicated on the X-axis. The black bars indicate the mean. The results were obtained using a one-way ANOVA, followed by the Tukey-Kramer test for multiple comparisons (* *P* < 0.05, ** *P* < 0.01)

To determine whether the complement activity was affected in CNVR_221 calves, the complement activity was examined at postnatal day 7 to exclude as much as possible the effects of environmental factors after birth. The average complement activity in CNVR_221 homozygotes at postnatal day 7 was 0.7- and 0.75-fold lower than that of wild animals (*P* < 0.01) and heterozygotes (*P* < 0.05), respectively (Fig. 4). The assembly of the C1s-C1r-C1r-C1s involves Ca^2+^-dependent C1r-C1s interactions between the CUB-EGF-CUB modules [28, 37–39]. Although *C1RL* (*LOC112446756*) only has CUB-EGF-CUB modules (Additional file 2: Table S4), the protein could affect the assembly of the complement complex via an unknown mechanism. These findings suggest that the complement activity in CNVR_221 homozygous calves slightly decreases and might lead to susceptibility to disease and infection. As the effect of the loss of *C1RL* (*LOC112446756*) on the complement activity is currently unknown, further research into the functions of *C1RL* (*LOC112446756*) on complement activity is required.

### The frequency of CNVR_221 in dead calves that suffered from infectious disease

To determine the CNVR_221 associated with infectious disease and calf mortality, we genotyped 246 dead calves that had suffered from diarrhea or pneumonia, respectively (Table S5), and 287 healthy cows that had given birth at least once, as control animals. To evaluate the population stratification, principal component analysis (PCA) was applied to the numerator relationship matrix of dead calves and healthy cows. The PCA plot showed no obvious distinction between the dead calves and the healthy cows (Additional file 4), suggesting that there was no population structure and none of the tests led to a bias for the genotyping analysis. The frequency of CNVR_221 in dead calves that suffered from an infectious disease and in healthy cows was 0.35 and 0.28, respectively (odds ratio = 2.2, Table 2). Additionally, we detected an excess of CNVR_221 in dead calves compared to healthy cows (*P* = 0.016; two-sided Fisher’s exact test, Table 2), suggesting that CNVR_221 was associated with mortality in Japanese Black calves suffering from an infectious disease. Notably, the CNVR_221 homozygous healthy cows were found in the population at a frequency of 0.08 (Table 2), indicating that the homozygote had survival ability under certain conditions; thus, CNVR_221 could be a quantitative trait locus (QTL) affecting the susceptibility of infected calves to death. In addition to the classical pathway, the complement pathway is also activated throughout lectin and alternative pathways, as reviewed in [33]; thus, some individuals with classical complement pathway deficiency could be rescued from infectious diseases by other complement pathways. As it is not known whether a slight reduction in the complement pathway in CNVR_221 homozygotes is directly connected to the onset of diarrhea or pneumonia, further investigation into the relationship between the complement activity and the CNVR_221 genotype, and the pathogenesis of infectious disease in calves will be required in many cases.

**Table 2:** Genotype frequencies of CNVR_221 in dead calves with infectious disease and healthy cows

	Dead calves (*N* = 246)^a^				Cows (*N* = 287)^b^			
Genotype	No.	Genotype freq	Exp No.	Exp freq	No.	Genotype freq	Exp No.	Exp freq
wild/wild	112	0.46	104.1	0.42	148	0.52	148.6	0.52
wild/CNVR_221	96	0.39	111.9	0.45	117	0.41	115.9	0.40
CNVR_221/CNVR_221	38	0.15	30.1	0.12	22	0.08	22.6	0.08
	CNVR_221 allele freq = 0.35				CNVR_221 allele freq = 0.28			

^a^Of the dead calves, 191 had diarrhea and 55 pneumonia (Additional file 2: Table S5). ^b^Healthy cows had given birth at least once. The association between CNVR_221 and health status was evaluated using the two-sided Fisher's exact test (*P* = 0.016)

## Conclusions

In this study, we evaluated calf mortality as a categorical trait with a threshold model and searched the CNVs associated with calf mortality in Japanese Black cattle. We identified a deleted-type CNVR, with decreasing complement activity, which was associated with calf mortality and susceptibility to infection. The mortality rate of Japanese Black calves during the first 180 days was 3.3%, which was mainly attributed to gastrointestinal and respiratory infectious diseases. Thus, CNVR could serve as a useful marker of the mortality of Japanese Black calves suffering from infections.

## Methods

### Ethical statement

All animal experiments were conducted according to the guidelines for the care and use of laboratory animals of the Shirakawa Institute of Animal Genetics, Northern Center of Agricultural Technology, General Technological Center of Hyogo Prefecture for Agriculture, and the University of the Ryukyus (A2018128). We obtained written agreement from the cattle farms to use the samples and data. The husbandry conditions of the animals were according to animal welfare guidelines in Japan for beef cattle [40]. A total of 44,001 Japanese Black cattle were used in this study, which were obtained from the Shirakawa Institute of Animal Genetics, Okayama University, and Hokubu Agricultural Technology Institute. After collecting the blood samples, the animals were released and retained in the farms.

### Phenotypic data and breeding value of calf mortality

The data consisted of 40,412 records of animals [18]. The threshold statistical model for calf mortality included the fixed effects of sex, parity, breeding farm, and combination of mating year and month as covariances:

$$ {U}_{ijkl}=\mu +{SEX}_i+{PARITY}_j+{FARM}_k+{YM}_l+{a}_{ijkl}+{e}_{ijkl} $$

$$ {Y}_{ijkl}=0\left({U}_{ijkl}\le t\right),{Y}_{ijkl}=1\left({U}_{ijkl}>t\right) $$

where *U*_*ijkl*_ is an unobservable underlying continuous variable (liability), *μ* is the overall mean, SEX_*i*_ is the fixed effect of sex, *PARITY*_*j*_ is the fixed effect of parity, *FARM*_*k*_ is the fixed effect of farm, *YM*_*l*_ is the fixed effect of month and year of birth, *a*_*ijkl*_ is the direct additive genetic effect as a random effect, and *e*_*ijkl*_ is the residual effect. *Y*_*ijkl*_ is the observation _*ijkl*_ for calf mortality; the trait was treated as a categorical trait: if the calf died, it was assigned a value of 2; if the cow lived it was assigned a value of 1. The observed binary response was assigned values of 1 and 0 if the liability was above a fixed threshold (t) and below *t*, respectively. Genetic parameters were estimated using the Gibbs sampling procedure. The THRGIBBS1F90 program was used to fit a threshold animal model for embryonic mortality [19, 20].

### SNP genotyping and CNVR detection

Blood was collected from 791 Japanese Black cows. Genomic DNA was extracted using the Easy-DNA kit (Invitrogen, Cat. #K1800–01). All samples were genotyped using the BovineHD BeadChip Array (Illumina, Cat. #WG-450-1002) [41]. SNPs genotype calling were performed using GenomeStudio V2011 (Illumina, version 1.9.4). The ARS-UCD1.2 assembly was used to map the SNP position [42]. CNVR was detected as previously reported [15], using PennCNV software (version June 2011) [43].

### Genome-wide CNV association study for calf mortality

Among the 861 CNVRs, 116 deleted-type CNVRs (Additional file 2: Table S1), which constituted a minor allele frequency greater than 0.01 in 1481 animals [15] (Additional file 2: Table S1), were used in the association study. Association study was performed on the breeding value of calf mortality of 791 cows using GEMMA software [27]. SNP association analyses were performed for 791 samples using SNPs in the BovineHD BeadChip Array. The estimated power in the genome-wide association study (GWAS) was 0.88 (Sample size = 791, Heritability = 0.165 [18], a= 0.000431, variance of the off-diagonals of the grm = 0.0026) using GCTA-GREML Power Calculator [44].

### Whole genome resequencing

Genomic DNA was sheared using a DNA shearing system (Covaris, Cat. #S220) prior to library preparation. Genomic DNA (200 ng) from six Japanese Black cattle (one wild/wild homozygote and five CNVR_221/CNVR_221 homozygotes) was used to generate sequence libraries, which were prepared using TruSeq Nano DNA library prep kit (Illumina, Cat. #20015964) and IDT for Illumina TruSeq DNA UD Indexes (Illumina, Cat. # 20020590) according to the manufacturer’s instructions. The sequence data were generated as 2 × 101 bp reads using the NovaSeq 6000 SP Reagent Kit (Illumina, Cat. # 20027465) and Novaseq6000, and processing and base calling were performed using Illumina Real-Time Analysis 3.

The sequence reads (fastq file) for each animal were aligned to ARS-UCD1.2 genome assembly [42] using bwa-0.7.17 [45]. The bam files were created using Samtools [46, 47], and duplicate reads were removed using Picard [48]. The realignment around indels and recalibration were performed using GATK-4.0.12.0 [49]. SNPs and indels were called using Haplotype caller by GATK. The data from the WGS were deposited in the Wagyu genome database (WGDB) of the Japan Livestock Technology Association (Yushima, Bunyouku, Tokyo 113–0034, Japan) and were managed by the WGDB consortium.

### Real-time quantitative PCR validation of CNVR

For real-time quantitative PCR (qPCR), we extracted genomic DNA from the blood of 28 Japanese Black cattle (two wild/wild homozygotes, 13 wild/CNVR_221 heterozygotes, and 13 CNVR_221/CNVR_221 homozygotes). Real-time qPCR was performed for CNVR validation using an ABI StepOnePlus Real Time PCR system (Applied Biosystems, Cat. #4376600). Primers and probes were designed for CNVR_221 (Additional file 2: Table S6). The basic transcription factor 3 gene (*BTF3*), which served as an internal qPCR standard for both copies at a locus (2n) [15, 50].

### Expression analysis using qPCR

We collected the livers of Japanese Black cattle (five wild/wild homozygotes, seven wild/CNVR_221 heterozygotes, and seven CNVR_221/CNVR_221 homozygotes) from a slaughterhouse (Hyogo prefecture). For RT qPCR, we isolated the total RNA from the livers using RNeasy mini kits (QIAGEN, Cat. #74104). cDNA was synthesized from 50 ng RNA using ReverTra Ace-α with random primers (TOYOBO Cat. #FSK-101). Bovine *C1RL* (*LOC112446756*) and *C1R* were amplified using the primers described in Additional file 1: Table S7. PCR products using the primer pairs for *C1RL* (*LOC112446756*) and *C1R* were cloned into the pCRII-TOPO vector (Invitrogen, Cat. #K460040), and the sequences were confirmed using M13_R primers. Real-time PCR was performed on an ABI StepOnePlus Real Time PCR system using the comparative Ct method with *glyceraldehyde-3-phosphate dehydrogenase* (*GAPD*) as the internal control.

### Measurement of complement activity

To determine whether the complement activities were affected in CNVR_221 calves, blood sera were collected postnatally (day 7) from 25 Japanese Black cattle (five wild/wild homozygotes, six wild/CNVR_221 heterozygotes, and 14 CNVR_221/CNVR_221 homozygotes). The sera were stored at 80 °C until the assay was performed. The 50% hemolytic complement activity [51] was measured in the serum using a complement activity assay kit (Denka-Seiken, Cat. #CH50) according to the manufacturer’s instructions. Hemolytic sheep erythrocytes, which were pre-coated with anti-sheep erythrocyte antibody, were measured at 541 nm with a plate spectrometer (BioRad, Cat. #iMark).

### CNVR_221 frequency in the Japanese Black cattle population

For the surveillance of the allele frequency, we randomly selected 246 dead calves that had suffered from diarrhea or pneumonia, as diagnosed by veterinarians. We used 287 healthy cows that had given birth at least once as control animals. To evaluate the population stratification, PCA was applied to the numerator relationship matrix. Real-time qPCR was performed for CNVR_221 genotyping using an ABI StepOnePlus Real Time PCR system. Primers and probes were designed for CNVR_221 (Additional file 2: Table S6).

## Supplementary Information

**Additional file 1:.** The estimated breeding value (EBV) distribution of 791 cows’ 1–180-day-old calves that died

**Additional file 2: Table S1** Features of deleted-type CNVRs on autosomes in this study. Positions are based on the ARS-UCD1.2 assembly of the bovine genome. NA: 12 SNPs in BovineHD beadchip (UMD3.1 assembly) were not assigned by the ARS-UCD1.2 assembly. **Table S2** Features of CNVR_221 and the surrounding SNPs. The SNP positions are based on the ARS-UCD1.2 assembly of the bovine genome. **Table S3** Detailed features of genes in CNVR_221 and in close proximity to CNVR_221. The SNP positions are based on the ARS-UCD1.2 assembly of the bovine genome. **Table S4** Detailed features of the domain of complement C1-related protein in CNVR_221 and in close proximity to CNVR_221. Domains were determined by the SMART protein sequence analysis tool. **Table S5** Descriptive statistics of dead calves that suffered from diarrhea or pneumonia. **Table S6** Primers and probes for CNVR_221 and BTF3. The SNP positions are based on the ARS-UCD1.2 assembly of the bovine genome. **Table S7** Primers and probes for *C1R subcomponent-like* and *C1R*.

**Additional file 3: **RNAseq data of the *C1RL* transcript (LOC112446756, XM_024992686.1) from National Center for Biotechnology Information *Bos taurus* Annotation release 106. Available from URL:https://www.ncbi.nlm.nih.gov/gene/?term=XM_024992686.1

**Additional file 4:.** Principal component analysis of dead calves and healthy cows. The first twenty eigenvectors and their values were calculated using an estimated numerator relationship matrix from 246 dead calves (magenta) and 287 healthy cows (blue). Scatter plots of PC1 vs. PC2 (A) and PC1 vs. PC3 (B).

## Data Availability

The data sets supporting the results of this article are included within the article and its additional file. The data from WGS that support the findings of this study are available from the Wagyu genome database (WGDB) of the Japan Livestock Technology Association (Yushima, Bunyouku, Tokyo 113–0034, Japan) and were managed by the WGDB consortium. Data are available from the authors upon reasonable request and with permission from WGDB. Refseq ID and protein name are based on Reference Sequence Database (RefSeq) of the National Center of Biotechnology Information (NCBI) (https://www.ncbi.nlm.nih.gov/refseq/).

## Abbreviations

CNV: Copy number variation
CNVR: CNV region
grm: Genetic relationship matrix
GWAS: Genome-wide association study
LD: Linkage disequilibrium
MAC: Membrane attack complex
PCA: Principal component analysis
PCR: Polymerase chain reaction
SNP: Single nucleotide polymorphism
