# Supplementary figures and images for: A 44-kb deleted-type copy number variation is associated with decreasing complement component activity and calf mortality in Japanese Black cattle
Source: BMC Genomics. 2021 Feb 6;22:107. doi: 10.1186/s12864-021-07415-6 (PMC7866702; doi:10.1186/s12864-021-07415-6)

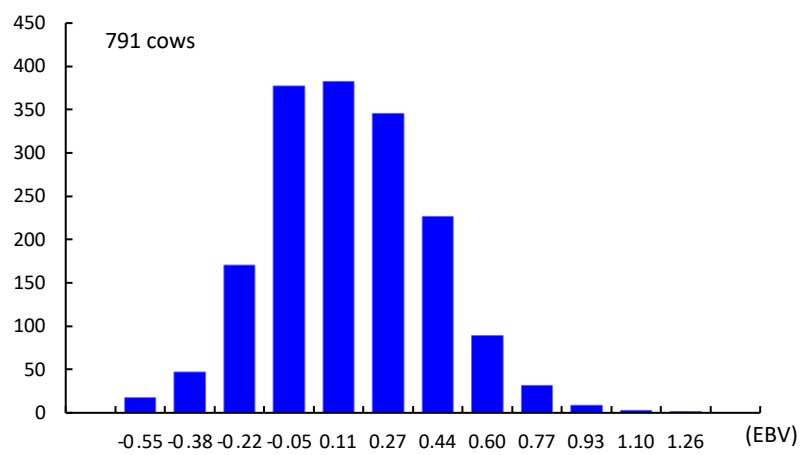

Supplement: Supplementary file 1 — Additional file 1:. The estimated breeding value (EBV) distribution of 791 cows’ 1–180-day-old calves that died [file 12864_2021_7415_MOESM1_ESM.pdf]

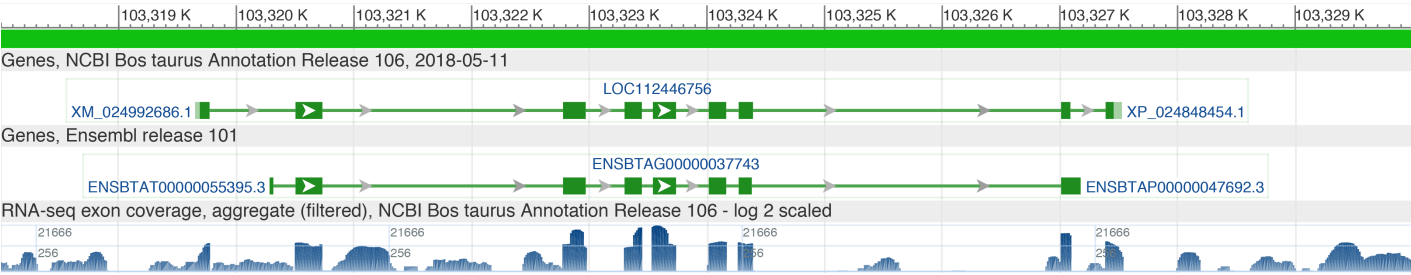

Supplement: Supplementary file 3 — Additional file 3: RNAseq data of the C1RL transcript (LOC112446756, XM_024992686.1) from National Center for Biotechnology Information Bos taurus Annotation release 106. Available from URL:https://www.ncbi.nlm.nih.gov/gene/?term=XM_024992686.1 [file 12864_2021_7415_MOESM3_ESM.pdf]

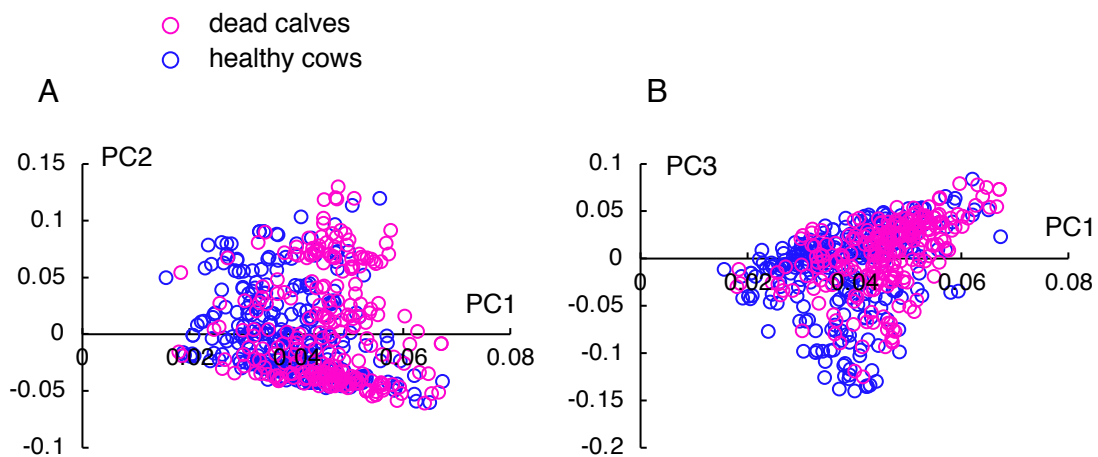

Supplement: Supplementary file 4 — Additional file 4:. Principal component analysis of dead calves and healthy cows. The first twenty eigenvectors and their values were calculated using an estimated numerator relationship matrix from 246 dead calves (magenta) and 287 healthy cows (blue). Scatter plots of PC1 vs. PC2 (A) and PC1 vs. PC3 (B). [file 12864_2021_7415_MOESM4_ESM.pdf]
